# Development and Quality Assurance of Multileaf Collimator (MLC) Auto-Feathering Junctions for Multi-Isocenter Supine Volumetric Modulated Arc Therapy (VMAT) Craniospinal Axis Irradiation on Halcyon

**DOI:** 10.7759/cureus.45640

**Published:** 2023-09-20

**Authors:** Joshua Misa, Shane McCarthy, William St. Clair, Damodar Pokhrel

**Affiliations:** 1 Department of Radiation Medicine, University of Kentucky, Lexington, USA

**Keywords:** mlc auto-feathering, patient-specific qa, rapidarc, vmat csi, halcyon

## Abstract

Currently, there is a lack of methods and tools that efficiently evaluate the auto-feathering junctions created by multileaf collimator (MLCs) for supine volumetric modulated arc therapy (VMAT) craniospinal irradiation (CSI) plans. We have investigated the feasibility of stitching together multi-isocenter fluence maps to then analyze the feathered junctions for patient-specific quality assurance (QA). Furthermore, we investigated the capability of Halcyon for the treatment of CSI patients. Three patients, who previously underwent VMAT CSI treatment on TrueBeam (6-MV flattening filter-free (FFF)) for 36 Gy in 20 fractions were replanned for Halcyon. A multi-isocenter approach with only translational superior-inferior shifts was used for both platforms. Each isocenter consists of two full arcs with anterior avoidance sectors, ±5° collimator rotations between arcs, and 5-8 cm of overlapping MLC auto-feathering junctions. All plans were QA’d via electronic portal imaging device (EPID) portal dosimetry and analyzed with a gamma criteria of 3%/3 mm. A variety of plan quality metrics were analyzed to evaluate dose distributions to the target, doses to organs at risk (OARs), and integral dose to the patient. A MATLAB script was developed to stitch the calculated and measured fluence maps in order to perform patient-specific QA for the composite fluence. The Halcyon plans provided highly conformal and homogenous dose distributions to the entire CSI target, superior to the clinical TrueBeam plans, while sparing critical organs with significantly lower values of V10Gy and V18Gy by up to 2% and 2.5%, respectively. Qualitative depictions of vertical dose profiles from the stitched DICOM of the entire CSI target for both planned and delivered fluence maps demonstrated equivalency, with slightly lower average pass rates with Halcyon (97%) compared to TrueBeam (99.9%). This approach to stitch multiple measured versus calculated EPID fluence maps has shown to be a feasible and accurate method and will be helpful for comprehensive VMAT CSI QA on both platforms. Further implementation of this script will be used in examining dosimetric impacts of daily patient positioning errors at MLC auto-feathering junctions.

## Introduction

Medulloblastomas are primitive cerebellar tumors of neuroectodermal origin. Approximately 20-25% of primary central nervous system (CNS) tumors consist of medulloblastomas with a median age at diagnosis of five years old [1]. Craniospinal axis irradiation (CSI) is one of the primary methods for treating these tumors. However, CSI is one of the most challenging techniques in radiation therapy due to issues of achieving uniform dose to the entire brain and spinal axis while minimizing hot and cold spots at the field matching junctions.

Conventionally, CSI has been performed using a 3D conformal radiation therapy technique in which the patient is set up in a prone position. Typically, bilateral cranial fields with posterior-anterior (PA) spine fields are used to cover the entirety of the target; these are then field-matched using a beam divergence or skin gap method [2]. The prone position was initially favored by radiation oncologists as a means to confirm these craniospinal junctions by direct light field visualization. However, these field-matching methods are very sensitive to intra- and inter-fraction positional uncertainties, especially in the cranio-caudal direction, resulting in severe deviations from the prescription dose inside the entire CSI planning target volume (PTV) [3,4]. In addition, applying these complex field-matching junctional shifts results in prolonged treatment times, which can be very challenging for the treatment of children and adolescents. Another disadvantage of this technique for children is when there is a need for anesthesia for immobilization. During anesthesia, accessing and maintaining of the airway become especially difficult in the prone position.

Further innovations in CSI treatments have involved the use of intensity-modulated radiation therapy (IMRT) with feathering to improve the dose homogeneity at these junctions [5]. The use of IMRT to perform CSI treatments has been demonstrated to improve dose homogeneity and conformity to the target while minimizing dose to organs at risk (OARs) [5-11]. The same results are also true when implementing volumetric modulated arc therapy (VMAT) for the treatment of CSI. IMRT and VMAT use a more complex technique of feathering compared to conventional techniques. This leads to the aforementioned more homogenous dose distributions across the junctions. In addition, the more complex feathering techniques have been found to be more robust against patient positional inaccuracy on PTV coverage and homogeneity [7,9,12-16]. A potential downside seen with VMAT is the increased spread of low dose to the patient, increasing the integral dose being delivered.

Recently, Varian Medical Systems (Palo Alto, CA) has introduced a new jawless, single energy, ring delivery system (RDS), the Halcyon linear accelerator (LINAC). This novel RDS delivers a 6-MV flattening filter-free (FFF) with a maximum output rate of 800 MU/min, a considerably lower output rate than TrueBeam’s 1400 MU/min for its 6MV-FFF beam. The Halcyon 6MV-FFF demonstrates a lower mean energy of 1.3 MeV and a depth of maximum dose at 1.3 cm as opposed to TrueBeam’s 6MV-FFF demonstrating 1.4 MeV and 1.5 cm, respectively. Halcyon’s unique dual-layered stack and staggered multileaf collimator (MLC) design offers many advantages over the Millennium 120 MLC design found on TrueBeam. Halcyon MLCs offer an effective 5 mm MLC width at the isocenter and a reduced beam penumbra of 0.1 mm due to its improved focused tip design. It also achieves faster maximal leaf speed up to 5 cm/s at the plane of the isocenter. The stacked and staggered design results in an ultra-low leakage and transmission of less than 0.4%, compared to the Millennium MLC’s 1.5%. Lastly, Halcyon is equipped with a kilovoltage (kV) X-ray image guidance radiation therapy (IGRT) imaging system that contains several advanced features, including its fast kV cone-beam computed tomography (kV-CBCT) and novel iterative CBCT reconstruction algorithm for patient setup verification.

The fast and efficient one-step patient setup that Halcyon offers is one of the main advantages of investigating the implementation of treating complex CSI patients that requires multi-isocenter planning onto Halcyon. Conversely, Halcyon does not include a light field and has a maximum field size of 28 × 28 cm^2^. In addition, Halcyon offers translation but no rotational table correction and has no manual table shift option, so the localization accuracy of the entire craniospinal axis is of the highest importance. To combat this, a planning technique in which no junction shifts are utilized and the shifts between the isocenters are only along the longitudinal direction has been shown to be feasible and able to provide outstanding coverage with acceptable doses to OARs [17,18]. Compared with TrueBeam's VMAT plans, Halcyon demonstrated superiority in terms of lower OAR doses and lower dose spillage [19].

At the time of preparing this manuscript, there was no tool available that allows clinics to investigate the delivery accuracy at these auto-feathering junctions. Given the dose uncertainty in these junctions due to auto-feathering, the verification of dose homogeneity is paramount. We propose a tool that will stitch fluence maps of the entire craniospinal target obtained from the electronic portal imaging device (EPID) via portal dosimetry (PD) QA procedure to be compared with fluence maps obtained from Eclipse Treatment Planning System (TPS, Varian Medical Systems, Palo Alto, CA). Having stitched fluences allows analysis of the entire craniospinal delivery and investigation of auto-feathering regions. This tool creates an additional layer of QA confirmation for documentation at these MLC auto-feathering junctions for treatment delivery accuracy on both TrueBeam and Halcyon RDSs. We investigated the accuracy and feasibility of our QA tool with three consecutive CSI patients who were previously treated on TrueBeam and were then replanned on Halcyon.

## Technical report

Materials and methods

In this study, we retrospectively analyzed a total of three patients who underwent supine VMAT CSI treatment on TrueBeam (6MV-FFF) for 36 Gy delivered in 20 fractions. All of these clinical patients were planned in Eclipse TPS with the Acuros XB dose calculation engine. For comparison, these patients were then replanned on Halcyon (6MV-FFF) with a multi-isocenter approach with only translational superior-inferior shifts between each isocenter, similar to the original clinical plans. The target length and number of isocenter characteristics for this patient cohort are summarized in Table 1. Each of the isocenters for both plans contained two full arcs with anterior avoidance sectors and a ±5° collimator offset between arcs. Both plans allowed for a 5-8 cm field overlap at junction sites. The dose calculation algorithm, dose calculation grid size, convergence mode, and planning objective settings were identical to the TrueBeam plans. However, jaw tracking is not available for the Halcyon plans due to its jawless MLC field design. All the Halcyon plans were then normalized to achieve identical target coverage to the corresponding TrueBeam plans.

**Table 1:** VMAT CSI target size and number of isocenters used in this case report on both platforms. VMAT: volumetric modulated arc therapy; CSI: craniospinal irradiation

	Superior/inferior target length (cm)	# of isocenters used: TrueBeam vs. Halcyon
Patient 1	79.2	3 vs. 3
Patient 2	82.5	3 vs. 4
Patient 3	67.0	3 vs. 3

For each plan, the dose delivery accuracy was evaluated by performing patient-specific QA measurements using a portal dosimetry VMAT QA procedure that was already established in our clinic on both platforms. The delivered fluence maps were measured on the EPID. The EPID on TrueBeam consists of a 40 × 40 cm^2^ area with a 0.336 mm resolution. The EPID on Halcyon has a 43 × 43 cm^2^ area with the same resolution as TrueBeam. For the Halcyon plans, the calculated portal dose used an anisotropic analytical algorithm (AAA) calculation model compared to the TrueBeam plans that used a portal dose image prediction (PDIP) calculation model, as a part of the departmental procedure. The main differences between the two platforms is that PDIP is a 2D PD algorithm and AAA is a 3D dose algorithm. 

The stitching of the planned and delivered fluence maps from both LINACs were performed with a MATLAB script. Our MATLAB script imports each of the field's DICOM fluence map and rescales the pixel values into units of calibration unit. Then, it will add up the pixel values of all the fields corresponding to each isocenter into a single image matrix. Next, the script will calculate an overlap factor in units of number of pixels based on the longitudinal couch shifts between each isocenter. A new DICOM image will be created with dimensions based on each field's DICOM dimensions and the previously calculated overlap factor. Lastly, the script will populate this new DICOM image with each of the previously combined isocenter image matrices and will be exported as a single DICOM for comparison. There will be added uncertainties with these stitched fluence maps due to the interpolation of the calculated overlap factor in units of cm to pixels. This may have added positional uncertainty to our measurement. Each machine had separate hurdles to overcome in order to properly stitch their planned and delivered fluence maps. On Halcyon, because the EPID measurement was taken at a source-to-image-distance (SID) of 154 cm, the corresponding planned fluences were also projected at 154 cm. To fully analyze the overlapping field junctions, these fluence maps were back-projected onto the plane of the isocenter of 100 cm for Halcyon. A benefit of Halcyon fluences was that they were always measured in a 1:1 aspect ratio, but Truebeam’s fluence maps were never recorded in a 1:1 aspect ratio. Measurements taken on Truebeam’s EPID for two arcs for a single isocenter may not have the same aspect ratio. This may be due to Truebeam's EPID measurements being cropped before being transferred back to the record and verification system. However, the planned fluences always had the same image aspect ratio.

For the two platforms, the plans were dosimetrically compared via their target coverage, conformity and homogeneity index, dose-volume histogram (DVH) of critical organs, integral dose (V_10Gy_ and V_18Gy_), and total monitor units (MUs). Furthermore, the delivery accuracy of the stitched fluence maps was assessed using a clinical gamma criterion of 3%/3 mm with a 10% dose threshold. This gamma criterion is what we clinically use, so we chose to report QA results using this criteria. Superior-to-inferior dose profiles were also analyzed and graphed out from these stitched fluences to ensure equivalencies between the planned and delivered fluence maps for both machines. Moreover, to independently verify the CSI VMAT plans, patient-specific quality assurance of physics second MU check was performed using an in-house Monte Carlo (MC) code for both platforms.

Results

The plan quality between the two machines was evaluated using a variety of plan quality metrics. Table 2 contains the dosimetric statistics evaluated for the PTV and OARs for both machines. Halcyon was able to provide a similar PTV dose coverage and showed improvements in the plan conformity index and homogeneity index for all the three patients. Integral dose to the patient was calculated using V­_10Gy_ and V_18Gy_. Halcyon consistently showed lower values by 1% and 2.5%, 2% and 1.4%, and 1% and 1% for patients 1, 2, and 3, respectively. The doses to OARs were similar for both plans across all patients. Halcyon was able to meet all the dose constraints per our clinical CSI protocol. The largest change between the two machines' plans was the total delivered MUs for the jawless Halcyon plans, which increased by a factor of 2 to 3 in comparison to the TrueBeam plans.

**Table 2:** Comparison of the dosimetric plan quality parameters for PTV, OAR doses, integral doses of V10Gy and V18Gy using body contour, and total MU for all the three patients on both machines. Prescription dose of 36 Gy in 20 fractions. PTV: planning target volume; OARs: organs at risk; MU: monitor unit; HI: homogeneity index; CI: conformity index

		Patient 1		Patient 2		Patient 3	
	Parameter	TrueBeam	Halcyon	TrueBeam	Halcyon	TrueBeam	Halcyon
PTV	D_95%_ (Gy)	35.79	36.00	36.09	36.00	35.73	36.00
	D_10%_ (Gy)	38.84	38.55	38.80	39.10	38.11	38.09
	D_mean_ (Gy)	37.67	37.66	37.77	38.00	37.24	37.30
	D_2cc_ (Gy)	39.95	39.88	40.77	39.99	39.18	38.79
	V_95%_ (%)	99.30	99.50	99.40	98.70	99.70	99.20
	V_105%_ (%)	50.30	49.70	54.20	66.70	27.50	24.80
	V_110%_ (%)	0.40	0.20	1.70	1.00	0.00	0.00
	HI	1.088	1.077	1.108	1.091	1.079	1.062
	CI	0.94	1.01	1.07	1.04	0.98	1.00
Integral dose	V_10Gy_, V_18Gy_ (%)	26.2, 14.9	25.7, 12.5	36.6, 15.5	24.9, 14.3	15.3, 8.0	14.3, 7.8
Optics pathway	Max dose (Gy)	38.89	39.83	37.30	39.28	37.67	38.45
Parotids	D_mean_ (Gy)	13.97	14.00	16.94	14.05	10.91	8.26
Thyroid	D_mean_ (Gy)	15.81	9.36	17.96	15.40	11.09	9.78
Lung	D_mean_ (Gy)	6.97	6.85	7.44	5.63	4.19	4.50
Heart	D_mean_ (Gy)	6.97	6.24	9.64	10.84	5.45	4.66
Esophagus	D_mean_ (Gy)	17.23	10.14	24.97	26.04	13.89	11.87
Liver	D_mean_ (Gy)	4.65	6.91	5.52	5.42	3.45	4.16
Spleen	D_mean_ (Gy)	3.79	4.25	1.76	2.46	2.63	3.13
Kidneys	D_mean_ (Gy)	4.38	3.86	5.46	4.14	3.14	4.59
Bowels	D_mean_ (Gy)	5.45	6.25	7.18	6.00	3.92	3.31
Total MU		758	2348	830	1619	836	1604

**Figure 1:**
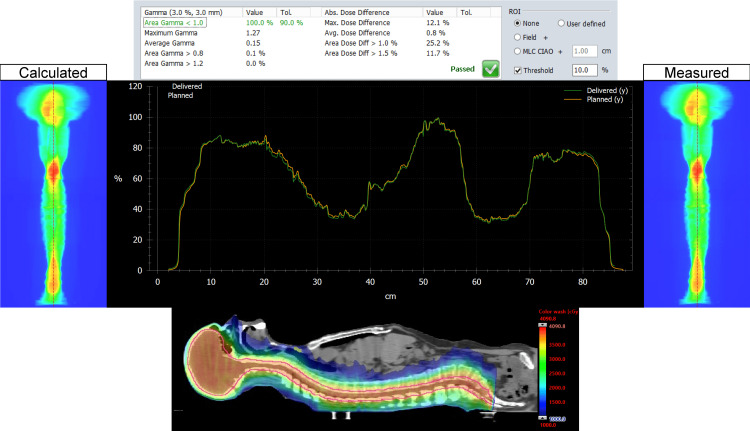
Bottom panel demonstrates the homogeneous dose distribution in the sagittal view (blue, 50% isodose colorwash) for the VMAT CSI patient #1 on TrueBeam. It includes the calculated vs. measured stitch fluence maps, vertical dose profiles superimposed to each other (middle panel), and PD QA gamma pass rate of 100% for the 3 mm/3% clinical criteria (top panel). VMAT: volumetric modulated arc therapy; CSI: craniospinal irradiation; PD QA: portal dosimetry quality assurance

**Figure 2:**
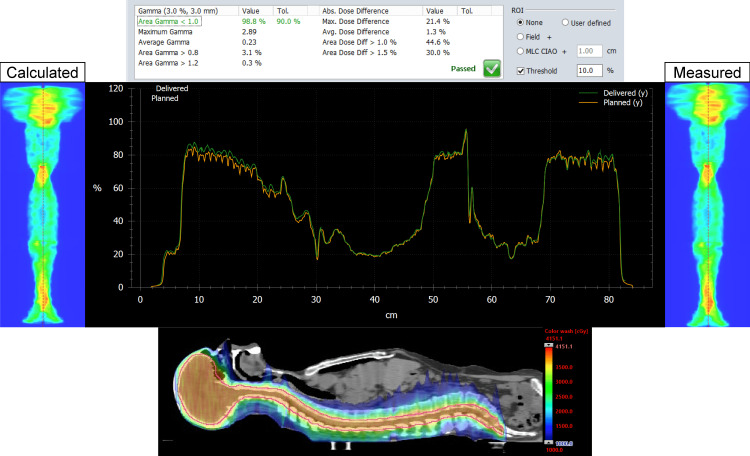
Bottom panel demonstrates the corresponding dose distribution (as shown in Figure 1) in the sagittal view (blue, 50% isodose colorwash) for the VMAT CSI patient #1 on Halcyon. It includes the calculated vs. measured stitch fluence maps, superimposed vertical dose profiles (middle panel) and the PD QA gamma pass rate of 98.8% for the 3 mm/3% clinical criteria (top panel). VMAT: volumetric modulated arc therapy; CSI: craniospinal irradiation; PD QA: portal dosimetry quality assurance

**Figure 3:**
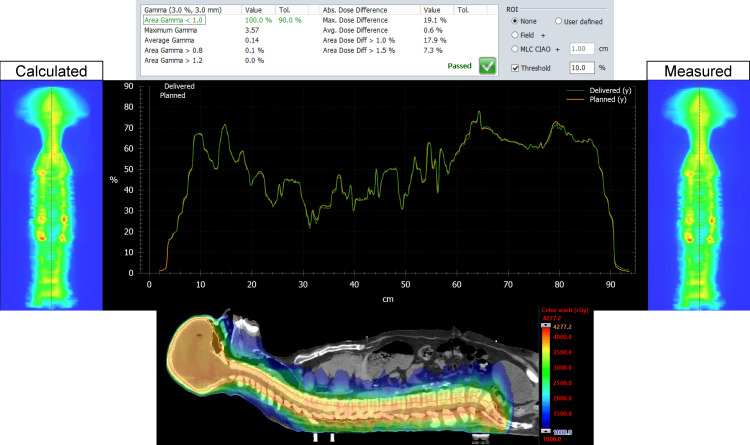
Bottom panel demonstrates the homogeneous dose distribution in the sagittal view (blue, 50% isodose colorwash) for the VMAT CSI patient #2 on TrueBeam. It includes the calculated vs. measured stitch fluence maps, vertical dose profiles superimposed to each other (middle panel), and PD QA gamma pass rate of 100% for the 3 mm/3% clinical criteria (top panel). VMAT: volumetric modulated arc therapy; CSI: craniospinal irradiation; PD QA: portal dosimetry quality assurance

**Figure 4:**
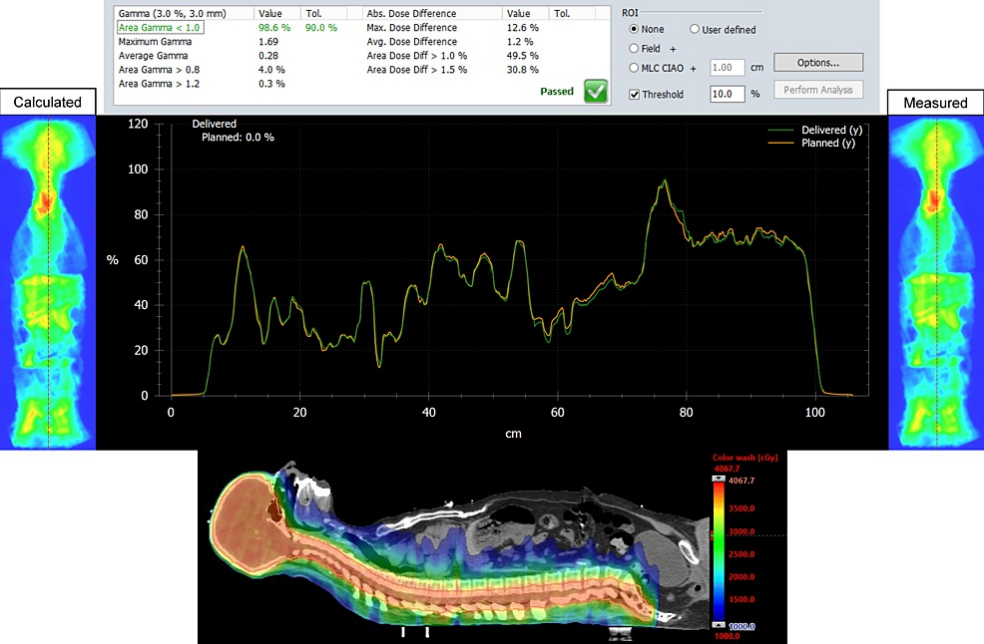
Bottom panel is the corresponding homogeneous dose distribution (as shown in Figure 3) in the sagittal view (blue, 50% isodose colorwash) for the VMAT CSI patient #2 on Halcyon. It includes the calculated vs. measured stitch fluence maps, vertical dose profiles superimposed to each other (middle panel), and PD QA gamma pass rate of 98.6% for the 3 mm/3% clinical criteria (top panel). VMAT: volumetric modulated arc therapy; CSI: craniospinal irradiation; PD QA: portal dosimetry quality assurance

**Figure 5:**
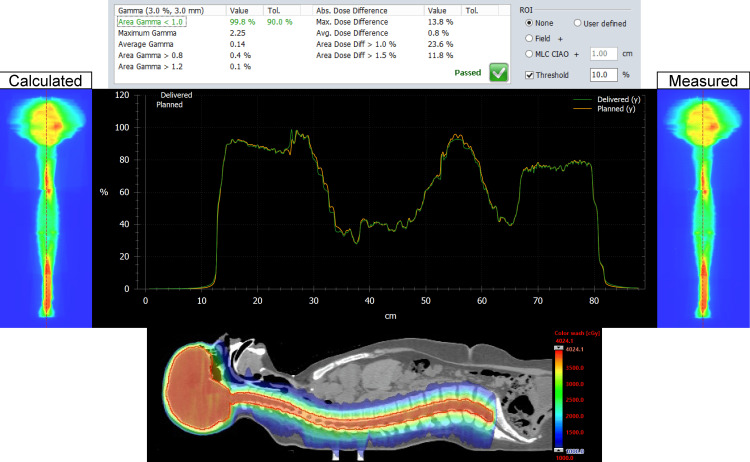
Bottom panel shows the homogeneous dose distribution in the sagittal view (blue, 50% isodose colorwash) for the VMAT CSI patient #3 on TrueBeam. It includes the calculated vs. measured stitch fluence maps, superimposed vertical dose profiles (middle panel), and PD QA gamma pass rate of 99.8% for the 3 mm/3% clinical criteria (top panel). VMAT: volumetric modulated arc therapy; CSI: craniospinal irradiation; PD QA: portal dosimetry quality assurance

**Figure 6:**
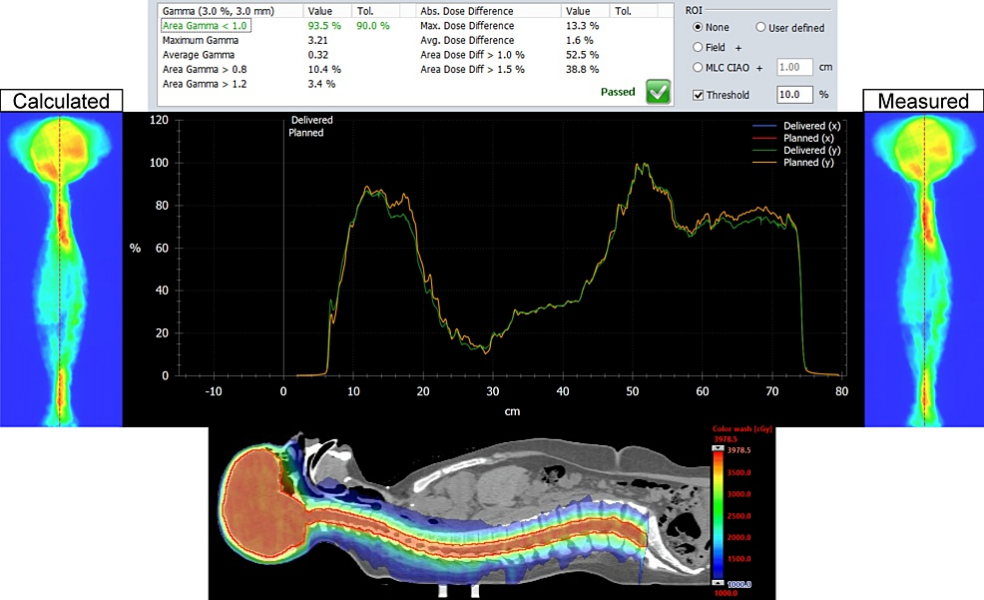
Bottom panel shows the corresponding homogeneous dose distribution (as shown in Figure 5) in the sagittal view (blue, 50% isodose colorwash) for the VMAT CSI patient #3 on Halcyon. It includes the calculated vs. measured stitch fluence maps, superimposed vertical dose profiles (middle panel), and PD QA clinical gamma pass rate of 93.5% for the 3 mm/3% clinical criteria (top panel). VMAT: volumetric modulated arc therapy; CSI: craniospinal irradiation; PD QA: portal dosimetry quality assurance

The MATLAB script was successful in stitching the planned and delivered fluence maps for both the Halcyon and TrueBeam plans for the entire CSI PTV. For each patient, on both plans, we analyzed the dose distribution in the Eclipse TPS, measured, and delivered fluence maps, vertical dose profile, and the resulting gamma analysis. Figures 1, 2 represent our results for patient 1. Figures 3, 4 show our results for patient 2. Lastly, Figures 5, 6 show the corresponding results for patient 3. Qualitatively comparing the vertical dose profiles of the planned and delivered fluences shows equivalencies demonstrating the efficacy of our proposed method. Comparing the dose distributions for all patients between the Halcyon and TrueBeam plans, we were able to achieve a similar target coverage and dose homogeneity across the entire target.

As show in Table 3, all the VMAT CSI patients exhibited higher QA pass rates on TrueBeam than on Halcyon, but both platforms achieved gamma passing rates greater than 90%, which is the criteria used in our clinic and is thus acceptable for treatment delivery. Per TG 218, we also reanalyzed using a gamma criteria of 3%/2 mm with a 10% threshold and still achieved a clinically acceptable passing rate of >90%. In addition, both platforms showed a high accuracy (average agreement within 4% for the TrueBeam plans vs. 2.6% for the Halcyon plans compared to the corresponding original Eclipse plans) for independent dose verification using an in-house MC algorithm for the second physics check.

**Table 3:** Tabulating the clinical gamma pass rates of patient-specific PD QA and independent in-house MC second physics check results for all the three example VMAT CSI cases on both platforms. VMAT: volumetric modulated arc therapy; CSI: craniospinal irradiation; PD QA: portal dosimetry quality assurance; MC: Monte Carlo

	PD QA (TrueBeam vs. Halcyon) (%)	MC second physics check (TrueBeam vs. Halcyon) (%)
Patient 1	100 vs. 98.8	0.6 vs. 1.0
Patient 2	100 vs. 98.6	5.5 vs. 3.8
Patient 3	99.8 vs. 93.5	5.9 vs. 3.0

## Discussion

The two main scopes of our study were to investigate the feasibility of treating multi-isocenter VMAT CSI patients on the limited-field-size Halcyon and to investigate the efficacy and feasibility of our stitching method as a QA tool. As discussed earlier, CSI treatments are very resource-intensive procedures on the side of planning, patient-specific QA, patient setup verification, and treatment delivery. Recent improvements in techniques, such as setting patients up in the supine position, use of IMRT auto-feathering at junctions, and VMAT, have greatly improved the clinical workflow while upholding plan quality and maintained patient safety. The ability to treat CSI patients on Halcyon will expand the access of curative treatments to an underserved patient cohort, including remote centers only equipped with Halcyon and underdeveloped nations. The current method of planning on Halcyon is to use a multi-isocenter approach (up to four in some cases) to overcome Halcyon’s limited field size and to have only superior/inferior shifts between isocenters to overcome Halcyon’s lack of rotational couch corrections. We utilized this multi-isocenter approach routinely in our clinic on the TrueBeam LINAC. Similar to our VMAT CSI results, comparisons between the Halcyon and TrueBeam plans have demonstrated identical dosimetric results as previous studies [17-19].

The dosimetry data shown in Table 2 supports our claim that Halcyon is feasible alternative to TrueBeam for the treatment of CSI patients. Replanning from TrueBeam to Halcyon, we were able to maintain similar plan metrics for the entire CSI target volume. Visually, this is confirmed when referring to Figures 1, 3, 5’s dose distributions on TrueBeam compared to the corresponding dose distributions planned on Halcyon RDS seen in Figures 2, 4, 6. Comparing the dose distributions also revealed that we can achieve comparable homogeneity and target conformity; this is reinforced when comparing the calculated conformity index between the two plans.

The Halcyon plans were able to achieve a sharper dose falloff, which indicates a lower dose spillage while maintaining a CI closer to 1.0. In addition, the Halcyon plans were able to achieve a lower value of homogeneity index (HI) than the TrueBeam plans. As previous studies have shown, Halcyon was able to provide an improvement in target dose homogeneity and conformity while also having a lower dose spillage [18,19]. This is further backed up when V­_10Gy_ and V_18Gy_ were analyzed in our study, in which Halcyon demonstrated lower values by up to 2.5%. This indicates that Halcyon can provide less integral dose to patient in comparison to treatments performed on TrueBeam. 

When analyzing doses to adjacent critical organs, neither machines showed superiority over the other for all OARs. Halcyon was able to meet the clinical requirements and guidelines for doses to OARs in the treatment of CSI patients established in our center. The drawback of Halcyon was seen when referring to the required larger total MU to be delivered. It is an increase of two to three times as much in comparison to the TrueBeam plans. Halcyon also has a maximum output rate of 800 MU/min as opposed to TrueBeam’s maximum output rate of 1400 MU/min. This does lead to longer beam on times for Halcyon, but due to the gantry rotation speed limitation of TrueBeam (typically at least one minute per revolution), Halcyon’s one-step patient setup and faster gantry rotation speeds (up to four revolutions per minute) may compensate for this and lead to a reduction in the overall treatment times. 

Referring to our results (Figures 1-6), we were successful in stitching the calculated and measured fluence maps and utilizing these stitched fluences in order to perform patient-specific QA as a combined map. For the three patients, there were no issues in the process of stitching or analyzing the stitched fluences. An important aspect to be taken away from these stitched fluences is the ability to qualitatively analyze CSI junctional sites that were MLC auto-feathered during the entire plan optimization. Included in the figures are vertical dose profiles taken at the middle of the target fluence maps. Although we saw a homogenous dose distribution in the 3D representation, the 2D PD QA representation does exhibit hot and cold regions. This is because homogenous 3D dose distributions on non-uniform volumes are being represented as a 2D fluence map at the EPID imager.

The analysis of the gamma results shows surprising results. When analyzing individual fields, the Halcyon fields were consistently showing higher pass rates than the TrueBeam plans. However, post-stitching of the fluence maps shows the opposite results. The TrueBeam plans for each patient demonstrated higher gamma passing rates than the stitched Halcyon fluence maps. Many factors may play into this discrepancy; one could be the difference in the calculation model used for the calculated fluence maps between the two machines. Another factor may be due to the differences in conditions in which the EPID measurements were taken. Halcyon has its EPID at a fixed SID of 154 cm, while the TrueBeam’s EPID measurements were taken at an SID of 100 cm. This may have led to parts of the MLC auto-feathering junctions to be cut off during measurements on the Halcyon RDS. The differences in SID may have also lead to Halcyon's EPID to not capture a lot of scattered low-energy photons at the edges of the radiation field. In addition, the portal dose calculation model we clinically used on Halcyon was an AAA model, while the one used for the TrueBeam plans is a PDIP model. The use of two different portal dose calculation models may attribute to the differences in gamma pass rates. However, this fluence map stitching tool has many potential applications in the use of PD-QA of CSI VMAT treatments. For the entire VMAT CSI plan, our in-house MC physics secondcheck calculation show accurate dose predication, including at MLC auto-feathering junctions within ±5% compared to the corresponding original Eclipse plans (for both platforms) for independent dose verification. Moreover, the independent dosimetric verification of physics second check of the MU per treatment field of the entire VMAT CSI plan can be done on both platforms via any commercially available second MU check software, such as RadCalc QA software (LAP, LifeLine Software, Inc., Austin, TX) or MU check (Oncology Data Systems, Inc., Oklahoma City, OK) or Mobious3D (Varian Medical Systems, Palo Alto, CA) within a few minutes, if the in-house second physics check system is not available.

There are several limitations to this dosimetric study and the analysis of our stitching tool. A main limitation is the limited number of patients that were used in this study, which is due to the limited number of CSI patient data available in our clinic. Another limitation of this study was that we did not cross-analyze our stitched fluence with the measurements of the dose distribution that do not need such stitching, such as film measurements. This will be useful in evaluating the effects of any interpolation that may have happened during the stitching process. Performing a cross-analysis between the two will further support the accuracy and utility of our tool. A future research direction that may be applicable is for it to be used in daily CBCT scans at different longitudinal table shifts on Halcyon to assist in the patient setup and verification as a combined daily CBCT scan for registration.

## Conclusions

If configured, Halcyon RDS has been shown to be a feasible alternative to the C-Arm TrueBeam LINAC in the treatment of supine CSI VMAT patients. Plan quality metrics comparing the two machines have revealed that limited-field-size Halcyon was able to provide a more homogenous and conformal dose distribution to the entire target, a similar dose to OAR, while offering lower integral doses to the patient. Furthermore, we successfully stitched the multi-isocenter fluence maps of the PD QA and demonstrated the combined patient-specific QA profile on these stitched fluences using our novel in-house script. This tool has potential to be very useful in examining the dosimetric impacts of daily patient positioning errors at MLC auto-feathering junctions in the future. Our results suggest that Halcyon may provide patient care for complex treatments, such as extended-large-field CSI treatments at remote community cancer centers or underserved patient cohort for clinics only equipped with Halcyon.

## References

[1] Kirsch DG, Tarbell NJ (2004). Conformal radiation therapy for childhood CNS tumors. Oncologist.

[2] Tatcher M, Glicksman AS (1989). Field matching considerations in craniospinal irradiation. Int J Radiat Oncol.

[3] Sarkar B, Munshi A, Manikandan A, Roy S, Ganesh T, Mohanti BK, Pradhan A (2018). A low gradient junction technique of craniospinal irradiation using volumetric-modulated arc therapy and its advantages over the conventional therapy. Cancer Radiother.

[4] Michalski JM, Klein EE, Gerber R (2002). Method to plan, administer, and verify supine craniospinal irradiation. J Appl Clin Med Phys.

[5] Studenski MT, Shen X, Yu Y (2013). Intensity-modulated radiation therapy and volumetric-modulated arc therapy for adult craniospinal irradiation--a comparison with traditional techniques. Med Dosim.

[6] Lee YK, Kim AT, Zhao P, Karotki A (2015). Practical dose delivery verification of craniospinal IMRT. J Appl Clin Med Phys.

[7] Pai Panandiker A, Ning H, Likhacheva A (2007). Craniospinal irradiation with spinal IMRT to improve target homogeneity. Int J Radiat Oncol Biol Phys.

[8] Seravalli E, Bosman M, Lassen-Ramshad Y (2018). Dosimetric comparison of five different techniques for craniospinal irradiation across 15 European centers: analysis on behalf of the SIOP-E-BTG (radiotherapy working group)(). Acta Oncol.

[9] Cao F, Ramaseshan R, Corns R (2012). A three-isocenter jagged-junction IMRT approach for craniospinal irradiation without beam edge matching for field junctions. Int J Radiat Oncol Biol Phys.

[10] Parker W, Filion E, Roberge D, Freeman CR (2007). Intensity-modulated radiotherapy for craniospinal irradiation: target volume considerations, dose constraints, and competing risks. Int J Radiat Oncol Biol Phys.

[11] Lee YK, Brooks CJ, Bedford JL, Warrington AP, Saran FH (2012). Development and evaluation of multiple isocentric volumetric modulated arc therapy technique for craniospinal axis radiotherapy planning. Int J Radiat Oncol Biol Phys.

[12] Myers P, Stathakis S, Mavroidis P, Esquivel C, Papanikolaou N (2013). Evaluation of localization errors for craniospinal axis irradiation delivery using volume modulated arc therapy and proposal of a technique to minimize such errors. Radiother Oncol.

[13] Neelakandan V, Christy S, Mukherji A, Reddy K (2018). Craniospinal irradiation by rapid Arc® technique in supine position: a dosimetric and clinical analysis. J Curr Oncol.

[14] Zhou Y, Ai Y, Han C, Zheng X, Yi J, Xie C, Jin X (2020). Impact of setup errors on multi-isocenter volumetric modulated arc therapy for craniospinal irradiation. J Appl Clin Med Phys.

[15] Wang K, Meng H, Chen J, Zhang W, Feng Y (2018). Plan quality and robustness in field junction region for craniospinal irradiation with VMAT. Phys Med.

[16] Chen J, Chen C, Atwood TF, Gibbs I, Soltys S, Fasola C, Xing L (2012). Volumetric modulated arc therapy planning method for supine craniospinal irradiation. J Radiat Oncol.

[17] Barsing S, Parab A, Singh A, Pemmaraju G (2022). Craniospinal irradiation by volumetric modulated arc therapy technique on halcyon. J Radiat Cancer Res.

[18] Stroubinis T, Psarras M, Zygogianni A, Protopapa M, Kouloulias V, Platoni K (2023). Craniospinal irradiation: a dosimetric comparison between O-ring LINAC and conventional C-arm LINAC. Adv Radiat Oncol.

[19] Sarkar B, Biswal SS, Shahid T (2023). Comparative dosimetric analysis of volumetric modulated arc therapy based craniospinal irradiation plans between Halcyon ring gantry and TrueBeam C-arm linear accelerator. Sci Rep.

